# Synthesis and Anticonvulsant Activity of Some Quinazolin-4-(3*H*)-one Derivatives

**DOI:** 10.3390/molecules13102557

**Published:** 2008-10-16

**Authors:** Hanan Georgey, Nagwa Abdel-Gawad, Safinaz Abbas

**Affiliations:** Pharmaceutical Chemistry Department, Faculty of Pharmacy, Cairo University, Kasr El-Eini Street, Cairo 11562, Egypt; E-mails: hamadido21@hotmail.com (N. A-G.), safi_esab@hotmail.com (S. A.) Tel.: 002-02-23639307; Fax: 002-02-23628426

**Keywords:** Quinazolin-4-(3*H*)-ones, piperazines, chloroacetamide, anticonvulsant activity

## Abstract

A number of 3-substituted-2-(substituted-phenoxymethyl) quinazolin-4(3*H*)-one derivatives have been synthesized. Their structures have been elucidated on the basis of elemental analyses and spectroscopic studies (IR, ^1^H-NMR, MS). A preliminary evaluation of the anticonvulsant properties of the prepared compounds has indicated that some of them exhibit moderate to significant activity, compared to a diazepam standard.

## Introduction

The quinazolin-4-(3*H*)-one ring system is considered an interesting moiety due to its wide ranging biological properties, which include antitumor [1,2,3], anti-HIV [4], selective estrogen beta modulator [5], anti-inflammatory [6,7,8], antibacterial [9,10,11,12], antidepressant [13] and CNS depressant activities [14,15,16,17,18]. The anticonvulsant activity was attributed to its ability to bind the noncompetitive site of α-amino-3-hydroxy-5-methyl-4-isoxazolepropionic acid (AMPA) receptors [16] 

**Figure 1:**
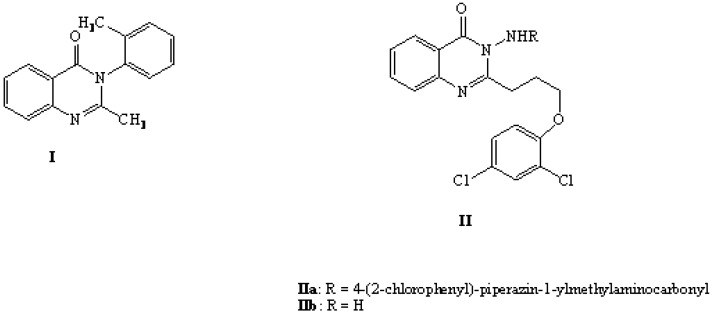
Known anticonvulsant compounds.

Since the discovery of methaqualone **I** (Figure 1) as a sedative hypnotic [19,20], the search for new anticonvulsant drugs with reduced toxicity and fewer side effects has been continuous. Our literature survey revealed that replacement of the methyl group by some other functionalities such as alkylthiomethyl or alkyloxymethyl groups reportedly yielded structural analogues which retained the anticonvulsant activity [21,22].

In a previous report [18], compounds **IIa,b** (Figure 1) were synthesized and tested for their anticonvulsant activity, which was comparable to that of diazepam. As a result, these compounds are potential leads for further design of more active compounds. In this investigation, side chain contraction of **II** with different pharmacophores groups was applied to prepare **4a,b**, **5a-c**, **6**, **7a-f**, **8a-d** and **9a,b**, in order to further study the effect of these moieties on the anticonvulsant activity.

## Results and Discussion

**Scheme 1:**
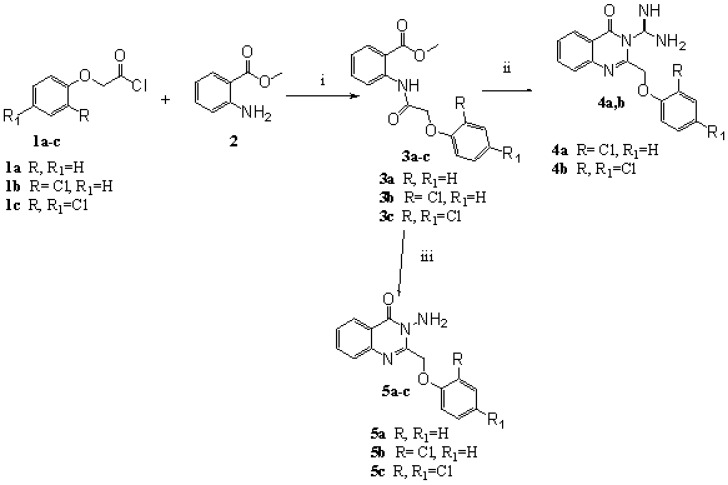
Synthetic Pathway for Compounds **3-5**.

The synthesis of the title compounds **4a,b**, **5a-c** was carried out as depicted in Scheme 1. Reaction of the un/substituted phenoxyacetyl chlorides **1a-c** with methyl anthranilate (**2**) in dry ether afforded the corresponding methyl 2-(2-(un/substituted phenoxy)acetamido)benzoates **3a-c**, which were reacted with guanidine hydrochloride in *n*-butanol to give the appropriate 2-(substituted phenoxymethyl)-4-oxoquinazolin-3(4*H*)-carboxamides **4a,b**. The structures of **4a,b** was established through spectroscopic (IR, ^1^H-NMR and mass) as well as elemental analyses data. The IR spectra showed the presence of NH and NH_2_ bands (3309-3186 cm^-1^), while the ^1^H-NMR spectra showed the disappearance of signals corresponding to the methyl ester protons and the presence of NH proton signals that disappeared on deuterium exchange. The mass spectrum of **4a** exhibited the molecular ion peak (M^+^ 328), in addition to fragments at *m/z* 285 [M–C(=NH)NH_2_] and 251 [M–C(=NH)NH_2_ and Cl]. Reaction of **3a-c** with hydrazine hydrate under previously described experimental conditions [18,25] yielded **5a-c**. The IR spectrum of **5a** showed a dramatic lowering in the carbonyl stretching band to 1681 cm^-1^, compared to the parent ester (1704 cm^-1^), as well as the appearance of two strong bands corresponding to asymmetric and symmetric NH_2_ stretching (3309, 3268 cm^-1^). The ^1^H-NMR spectrum lacked the methyl ester protons and displayed the amino protons (5.28 ppm), while the mass spectrum showed the molecular ion peak of the compound (*m/z* 267), and peaks at *m/z* 251(M–NH_2_) and 145 (M–NH_2_ and CH_2_OC_6_H_5_).

*Reaction conditions*: i) dry ether, ii) NH_2_C(NH)NH_2_·HCl, *n*-butanol, TEA, iii) NH_2_NH_2_·H_2_O, *n*-butanol

**Scheme 2:**
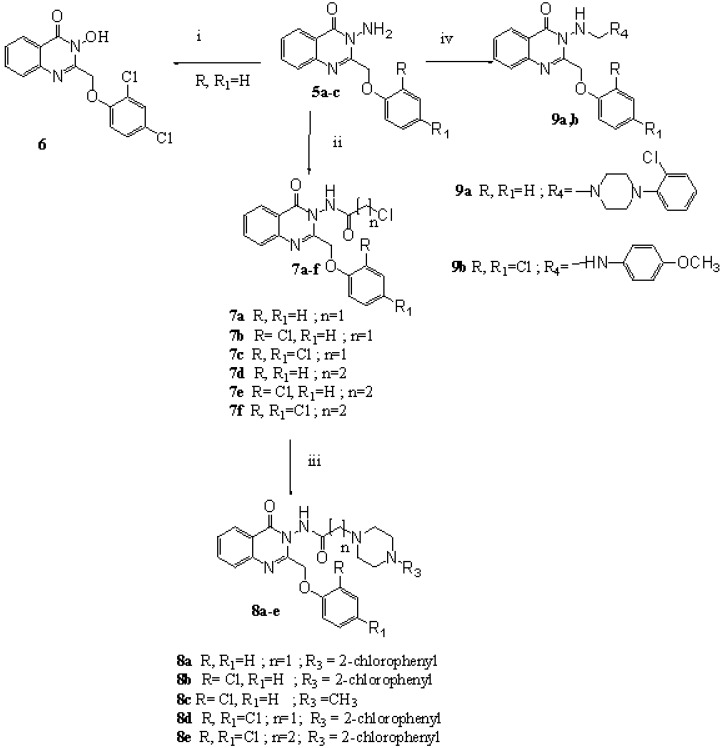
Synthetic Pathway for Compounds **6-9**.

Diazotization of **5c**, followed by hydrolysis, yielded the hydroxamic acid derivative **6** (Scheme 2). Comparing the spectral data (IR, ^1^H-NMR and mass spectra) of **5c** with **6** revealed that the two NH_2 _bands in the IR (3300, 3200 cm^-1^) and their corresponding NMR signal (4.78 ppm) [18] had completely vanished and instead, a broad OH group band (3423 cm^-1^) and its corresponding ^1^H-NMR signal (9.71 ppm) appeared. The mass spectrum showed the molecular ion peak of the compound (M^+^ 337), and peaks at *m/z* 285 (M – OH and Cl) and 251 (M – OH and 2Cl).

Reaction of **5a-c** with chloroacetylchloride in DMF gave 2-chloro-*N*-(4-oxo-2-(un/substituted phenoxymethyl)quinazolin-3(4*H*)-yl)acetamides **7a-c** (Scheme 2). Similarly, the reaction with chloropropionylchloride afforded **7d-f**. Reactions of **7a-c** with secondary amines (namely, 2-*N*-(chlorophenyl)piperazine and *N*-methylpiperazine) in dry acetonitrile in the presence of potassium carbonate yielded compounds **8a-d**, which showed upfield shifted acetamidomethylene protons (3.37-3.10), compared to the parent chloroacetamidomethylene function of **7a-c** (4.34-4.22 ppm). Similarly, reaction of **7f** with 2- chlorophenylpiperazine yielded **8e**. 

Also, **5** reacted with formaldehyde and amines (namely, 2-*N*-(chlorophenyl)piperazine and 4-methoxyaniline) in a Mannich reaction yielding **9a,b** (Scheme 2). The IR showed the disappearance of the NH_2_ bands together with the presence of NH band (3220 and 3250 cm^-1^ for **9a** and **9b**, respectively). The ^1^H-NMR of **9b** showed the presence of methoxy protons (3.78 ppm) and the methylene protons (1.7 ppm). 

*Reaction conditions*: i) NaNO_2_, HCl, hydrolysis, ii) Cl(CH_2_)_n_COCl, dry DMF, iii) , acetonitrile, K_2_CO_3_, iv) HCHO, substituted amines, dry DMF.

The newly synthesized compounds were screened for their anticonvulsant activity by the Maximal Electroshock (MES) induced seizures method [23,24], wherein electroshocks was applied via ear-lip electrodes using diazepam as a reference drug.

**Figure 2:**
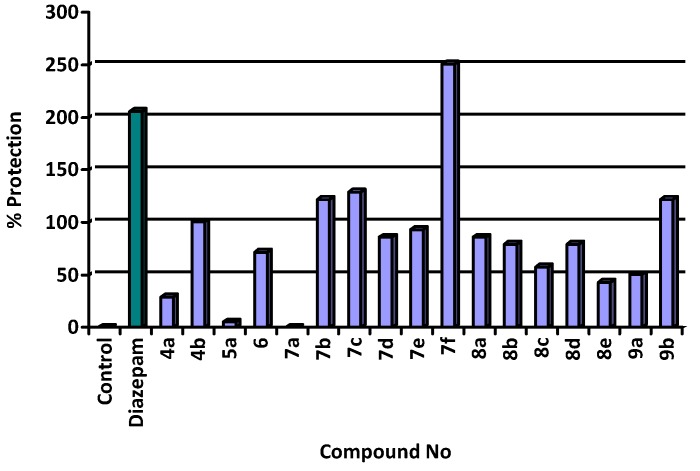
Graphical representation of the anticonvulsant activity of the quinazolin-4(3*H*)-ones compared to diazepam.

Data are presented in Table 1 to show the mean convulsion threshold, percentage protection and percentage potency for both the newly synthesized compounds and diazepam. The % protection is illustrated in Figure 2.

**Table 1:** Anticonvulsant activity of diazepam and the newly synthesized compounds.

Comp.	Mean convulsion threshold ± S.E	% Protection	% Potency
**Control**	2.33 ± 027	0	0
**Diazepam**	7.11 ± 0.33*	205.42*	--
**4a**	3.00 ± 0.27	28.76	42.19
**4b**	4.67 ± 0.27*	100.43*	65.68*
**5a**	2.45 ± 0.31	5.15	34.45
**6**	4.00 ± 0.41	71.67	56.25
**7a**	2.33 ± 0.14	0	0
**7b**	5.17 ± 0.50*	121.89*	72.71*
**7c**	5.33 ± 0.18*	128.76*	74.96*
**7d**	4.33 ± 0.45*	85.84*	60.90*
**7e**	4.50±0.20*	93.13*	63.29*
**7f**	8.17 ± 0.77*	250.64*	114.90*
**8a**	4.33 ± 0.41*	85.84*	60.90*
**8b**	4.17 ± 0.23	78.97	58.64
**8c**	3.67 ± 0.32	57.51	51.61
**8d**	4.17 ± 0.23	78.97	58.64
**8e**	3.33 ± 0.32	42.92	46.83
**9a**	3.50 ± 0.34	50.21	49.22
**9b**	5.17 ± 0.34*	121.89*	72.71*

Values represent means of six animals' ± standard error. * P≤ 0.05 statistically significant from control group (using Dunnett's test as post hoc test)

2-((2,4-Dichlorophenoxy)methyl)-4-oxoquinazolin-3(4*H*)-carboxamidine (**4b**) revealed significant activity, while its 3-amino- and 2-(2-chlorophenoxy) analogues (**5c** [18] and **4a**, respectively, are inactive. On the other hand, its 3-hydroxy analogue **6** showed mild activity.

Replacement of the 3-amino group by a 3-chloromethylcarbonylamino moiety as in **7a-c** leads to an increase in the activity of the 2-(2-chlorophenoxy) and 2-(2,4-dichlorophenoxy) substituted compounds **7b** and **7c** respectively. Moreover, the 3-(2-chloroethyl)carbonylamino derivatives **7d** and **7f** were found to be more active than their corresponding 3-chloromethylcarbonylamino analogues **7a** and **7c** respectively and the order of activity of compounds **7d-f** is: 2-(2,4-dichlorophenoxy) derivative **7f** > 2-(2-chlorophenoxy) derivative **7e** > 2-phenoxy derivative **7d**. With the exception of **8a**, addition of the 4-substituted piperazin-1-yl moiety to the chloroacetamido/propanamido derivatives **7a-f** to produce **8a-e** resulted in a decrease in the anticonvulsant activity. Besides, the 4-methylpiperazin-1-yl derivative is less active than the 4-(2-chlorophenyl)piperazin-1-yl analogue (c.f. compound **8c** and **8b**).

Finally, substitution of the 3-amino group with a 3-(4-(2-chlorophenyl)piperazin-1-yl)methylamino one, as in **9a** resulted in a mild increase in the activity, while substitution with 3-(4-methoxyphenyl)amino (compound **9b**) yielded a remarkable increase in the activity. This result indicated that replacement of the COCH_2_ group of **8a** by a CH_2_NH group as in **9a** decreases the activity. 

## Conclusions

Different quinazolin-4-(3*H*)-one derivatives **4a,b**, **5a**, **6**, **7a-f**, **8a-e** and **9a,b**, as well as methyl 2-(2-phenoxyacetamido) benzoate **3a** were synthesized, completely characterized and evaluated for their anticonvulsant activity. The test results showed that compounds **4b**, **7b-f**, **8a** and **9b** exhibited significant anticonvulsant activity while compounds **6**, **8b** and **8d** showed mild to moderate activity. 

Concerning the substitution in position 2 of quinazolin-4-(3*H*)-one derivatives, the order of activity was found to be 2-(2,4-dichlorophenoxy)>2-(2-chlorophenoxy)>2-phenoxy. Compound **8a** was an exception to this trend.

Additionally, substitution in position 3 of quinazolin-4-(3*H*)-one derivatives affected the biological activity. Thus, the 3-(2-chloroethyl)carbonylamino derivatives **7d** and **7f** are more active than their 3-chloromethylcarbonylamino analogues **7a** and **7c**. Also, 3-carboxamidino and 3- hydroxy derivatives are more active than their 3-amino analogue as in **4b**, **6** and **5c** respectively. Finally, the anticonvulsant activity of the tested compounds could be arranged in descending order as follows: **7f** > **7c** > **7b** ≈ **9b** > **4b** > **7e** > **7d** and **8a**. 

## Experimental

### General

TLC was perfomed on Fluka silica gel on aluminium TLC plates (0.2 mm thickness) with 254 nm fluorescent indicator using ethyl acetate-petroleum ether (5:5) or (6:4) as eluents. All melting points were determined by the open capillary tube method using an IA 9100MK-digital melting point apparatus and are uncorrected. IR spectra were recorded on a Bruker Vector 22 spectrophotometer. ^1^H-NMR spectra were recorded on a Varian Mercury VX- 300 NMR spectrometer and they were run at 300 MHz in deuterated chloroform (CDCl_3_) or dimethylsulfoxide (DMSO-d_6_); the chemical shifts were quoted in δ units and were related to that of the solvents. Mass spectra were recorded on Finnigan MAT, SSQ 7000, mass spectrometer at 70 eV. Elemental Microanalyses were carried out using Heraew and Vario EL III (elemntar), CHNS analyzer at the Microanalytical Center, Cairo University. Compounds **3b,c** [12] and **5b,c** [18] were prepared according to the reported methods, while compound **5a** was prepared by the method of Shishoo *et al*. [25] but using methyl 2-(2-phenoxyacetamido)benzoate (**3a**) and *n*-butanol as a solvent.

### Synthesis of methyl 2-(2-phenoxyacetamido)benzoate (**3a**):

A solution of 2-phenoxyacetylchloride (10 mmol) in dry ether (10 mL) was added dropwise to a cooled solution of methylanthranilate (15 mmol) in dry ether (50 mL). The reaction was stirred at room temperature (25-30 ^o^C) for 24 h and then filtered. The filtrate was extracted with dil. HCl (3 x 20 mL), washed with water, then extracted with sodium hydroxide (10 %, 3 x 20 mL) and finally washed with water. The organic layer was filtered over anhydrous sodium sulfate and evaporated; the remaining solid was crystallized from ethanol. Yellow crystals; m.p. 83-85 ^o^C; yield 83%; IR ν/cm^-1^: 3246, 1704, 1601, 1585, 1528; ^1^H-NMR δ/ppm (CDCl_3_): 4.07 (s, 3H, CH_3_), 4.77 (s, 2H, CH_2_O), 7.14-8.93 (m, 9H, arom. H), 12.21 (s, 1H, NH (D_2_O exchange)); Anal. calcd. for C_16_H_15_NO_4_ (285.29): C, 67.36; H, 5.30; N, 4.91%. Found: C, 67.14; H, 5.51; N, 4.64%. 

### Synthesis of 2-((substituted phenoxy)methyl)-4-oxoquinazolin-3(4H)-carboxamidines **4a,b**:

A mixture of equimolar amounts of guanidine hydrochloride and the corresponding **3b,c** (10 mmol) in n-butanol (20 mL) containing triethylamine (20 mmol), was refluxed for 12 h. The reaction mixture was evaporated and the residue crystallized from ethanol affording **4a, b**.

*2-((2-Chlorophenoxy)methyl)-4-oxoquinazolin-3(4H)-carboxamidine* (**4a**): White crystals; m.p. 61-62 ^o^C; yield 83%; IR ν/cm^-1^: 3309, 3268, 3186, 1681, 1601, 1580, 1550; ^1^H-NMR δ/ppm (CDCl_3_): 4.27 (s, 2H, NH_2_ (D_2_O exchange)), 4.70 (s, 2H, CH_2_O), 6.95-8.06 (m, 7H, arom. H), 8.73 (d, *J*=8.4 Hz, 1H, arom-H_5_), 11.92 (s, 1H, NH (D_2_O exchange)). Ms: m/z (%) 328 (M, 3), 327 (M-1, 63), 326 (M-2, 100), 285 (C_15_H_11_ClN_2_O_2_, 4), 251 (C_15_H_11_N_2_O_2_, 51); Anal. calcd. for C_16_H_13_ClN_4_O_2_ (328.75): C, 58.45; H, 3.99; N, 17.04%. Found: C, 58.50; H, 4.02; N, 17.16%. 

*2-((2,4-Dichlorophenoxy)methyl)-4-oxoquinazolin-3(4H)-carboxamidine* (**4b**): White crystals; m.p. 86-87 ^o^C; yield 89%; IR ν/cm^-1^: 3300, 3250, 1697, 1601, 1590, 1521; ^1^H-NMR δ/ppm (CDCl_3_): 4.28 (t, 2H, NH_2_ (D_2_O exchange)), 4.68 (s, 2H, CH_2_O), 6.90-8.03 (m, 6H, arom-H), 8.72 (d, *J*=5.8 Hz, 1H, arom-H_5_), 11.89 (s, 1H, NH (D_2_O exchange)); Anal. calcd. for C_16_H_12_Cl_2_N_4_O_2_ (363.36): C, 52.91; H, 3.32; N, 15.44%. Found: C, 52.45; H, 3.40; N, 15.14%. 

### Synthesis of 2-((2,4-dichlorophenoxy)methyl)-3-hydroxyquinazolin-4(3H)-one (**6**):

To a solution of **5c** (10 mmol) in 1N hydrochloric acid (20 mL), sodium nitrite solution (10%, 10 mL) was added while stirring in an ice bath. The reaction mixture was stirred for an hour then boiled for 5 minutes, cooled and extracted with methylene chloride (3 x 5 mL). The combined organic layers were collected and dried on anhydrous sodium sulfate. The solvent was removed under reduced pressure and the separated solid was crystallized from ethanol-chloroform (3:1) to give white crystals; m.p. 221-223 ^o^C; yield 71%; IR ν/cm^-1^: 3423, 1687, 1612, 1550; ^1^H-NMR δ/ppm (CDCl_3_): 5.14 (s, 2H, CH_2_O), 6.96-7.86 (m, 6H, arom-H), 8.31 (d, *J*=7.8 Hz, 1H, arom-H_5_), 9.71 (s, 1H, OH (D_2_O exchange)). Ms: m/z (%) 341 (M+4, 8), 339 (M+2, 13.5), 337 (M, 4), 285 (C_15_H_11_ClN_2_O_2_, 45), 251 (C_15_H_11_N_2_O_2_, 5), 69 (C_2_HN_2_O, 100); Anal. calcd. for C_15_H_10_Cl_2_N_2_O_3_ (337.15): C, 53.44; H, 2.99; N, 8.31%. Found: C, 53.50; H, 3.10; N, 8.50%. 

### Synthesis of 2/3-chloro-N-(4-oxo-2-(un/substituted phenoxymethyl)quinazolin-3(4H)-yl)acetamide/ propanamides **7a-f**:

A solution of the appropriate **5a-c** (5 mmol) in dry DMF (5 mL) containing chloroacetylchloride or chloropropionylchloride (5.5 mmol) was stirred at room temperature (25-30 ^o^C) for 24 h. The solution was poured onto crushed ice and the resulting solid was filtered, washed and crystallized from the appropriate solvent. 

*2-Chloro-N-(4-oxo-2-(phenoxymethyl)quinazolin-3(4H)-yl)acetamide* (**7a**): White crystals from ethanol; m.p. 150-151 ^o^C; yield 81%; IR ν/cm^-1^: 3290, 1730, 1685, 1600, 1560; ^1^H-NMR δ/ppm (CDCl_3_): 4.28 (d, *J*=4.14 Hz ,2H, CH_2_Cl), 5.07 (s, 2H, CH_2_O), 7.06-7.74 (m, 8H, arom-H), 8.25 (d, 1H, arom-H_5_, J=7.65), 9.15 (s, 1H, NH (D_2_O exchange)); Anal. calcd. for C_17_H_14_ClN_3_O_3_ (343.76): C, 59.40; H, 4.10; N, 12.22%. Found: C, 59.90; H, 4.36; N 12.24%. 

*2-Chloro-N-(2-((2-chlorophenoxymethyl)-4-oxo-quinazolin-3(4H)-yl)acetamide* (**7b**): White crystals from ethanol; m.p. 172-174 ^o^C; yield 78%; IR ν/cm^-1^: 3200, 1725, 1680, 1600, 1535; ^1^H-NMR δ/ppm (CDCl_3_): 4.27 (s, 2H, CH_2_Cl), 5.16 (s, 2H, CH_2_O), 7.02-7.99 (m, 7H, arom-H), 8.18 (d, *J*=6.84 Hz,1H, arom-H_5_), 10.10 (s, 1H, NH (D_2_O exchange)); Anal. calcd. for C_17_H_13_Cl_2_N_3_O_3_ (378.20): C, 53.99; H, 3.46; N, 11.11%. Found: C, 53.91; H, 3.52; N, 10.95%. 

*2-Chloro-N-(2-((2,4-dichlorophenoxymethyl)-4-oxo-quinazolin-3(4H)-yl)acetamide* (**7c**): White crystals from ethanol-chloroform (3:1); m.p. 186-187 ^o^C; yield 81%; IR ν/cm^-1^: 3200, 1715, 1680, 1600, 1570; ^1^H-NMR δ/ppm (CDCl_3_): 4.22 (d, *J*=14.6 Hz ,1H, upfield proton of CH_2_Cl), 4.34 (d, *J*=15.4 Hz, 1H, downfield proton of CH_2_Cl), 5.14 (s, 2H, CH_2_O), 7.09-7.86 (m, 6H, arom-H), 8.29 (d, *J*=8.0 Hz ,1H, arom-H_5_), 8.97 (s, 1H, NH (D_2_O exchange)). Ms: m/z (%) 415 (M+3, 1), 414 (M+2, 0.43), 413 (M+1, 2), 412 (M, 0.5), 411 (M-1, 2), 376 (C_17_H_12_Cl_2_N_3_O_3_, 38), 319 (C_15_H_10_Cl_2_N_3_O_2_, 4), 285 (C_15_H_11_ClN_2_O_2_, 7), 251 (C_15_H_11_N_2_O_2_, 14), 250 (100); Anal. calcd. for C_17_H_12_Cl_3_N_3_O_3_ (412.99): C, 49.48; H, 2.93; N, 10.18%. Found: C, 49.65; H, 3.00; N 10.10%.

*3-Chloro-N-(4-oxo-2-(phenoxymethyl)quinazolin-3(4H)-yl)propanamide* (**7d**): Colourless crystals from ethanol; m.p. 138-140 ^o^C; yield 69%; IR ν/cm^-1^: 3290, 1720, 1680, 1600, 1550; ^1^H-NMR δ/ppm (CDCl_3_): 2.85 (t, *J*=7.2 Hz, 2H, CH_2_Cl), 3.78 (d, *J*=5.8 Hz, 2H, CH_2_CO), 5.12 (s, 2H, CH_2_O), 6.96-7.74 (m, 8H, arom-H), 8.17 (d, *J*=8.0 Hz, 1H, arom-H5), 8.83 (s, 1H, NH (D_2_O exchange)). Ms: m/z (%) 358 (M+1, 5), 356 (M-1, 15), 263 (100); Anal. calcd. for C_18_H_16_ClN_3_O_3_ (357.79): C, 60.42; H, 4.51; N, 11.74%. Found: C, 60.68; H, 4.78; N, 11.31%.

*3-Chloro-N-(2-((2-chlorophenoxymethyl)-4-oxo-quinazolin-3(4H)-yl)propanamide* (**7e**): White crystals from ethanol; m.p. 154-155 ^o^C; yield 70%; IR ν/cm^-1^: 3200, 1710, 1680, 1600, 1520; ^1^H-NMR δ/ppm (CDCl_3_): 2.91(m, 2H, CH_2_Cl), 3.77(m, 2H, CH_2_CO), 5.08 (d, *J*=12.4 Hz,1H, upfield proton of CH_2_O), 5.16 (d, *J*=12.6 Hz, 1H, downfield proton of CH_2_O), 6.95-7.75 (m, 7H, arom-H), 8.25 (d, 1H, arom-H_5_, J=7.8), 8.70 (s, 1H, NH (D_2_O exchange)); Anal. calcd. for C_18_H_15_Cl_2_N_3_O_3_ (392.23): C, 55.10; H, 3.85; N, 10.71%. Found: C, 55.24; H, 4.00; N 10.68%.

*3-Chloro-N-(2-((2,4-dichlorophenoxymethyl)-4-oxo-quinazolin-3(4H)-yl)propanamide* (**7f**): White crystals from ethanol-chloroform (3:1); m.p. 208-209 ^o^C; yield 63%; IR ν/cm^-1^: 3220, 1710, 1670, 1600, 1580; ^1^H-NMR δ/ppm (CDCl_3_): 2.91 (m, 2H, CH_2_Cl), 3.90 (m, 2H, CH_2_CO), 5.12 (d, *J*=12.4 Hz, 1H, upfield proton of CH_2_O), 5.21 (d, *J*=12.6 Hz, 1H, downfield proton of CH_2_O), 7.05-7.82 (m, 6H, arom-H), 8.26 (d, *J*=7.8 Hz,1H, arom-H_5_), 8.30 (s, 1H, NH (D_2_O exchange)); Anal. calcd. for C_18_H_14_Cl_3_N_3_O_3_ (426.68): C, 50.67; H, 3.31; N, 9.85%. Found: C, 50.81; H, 3.20; N, 9.84%.

### Synthesis of 2/3-(4-substituted piperazin-1-yl)-N-(4-oxo-2-(un/substituted phenoxymethyl)quinazolin-3(4H)-yl)acetamide/propamides **8a-e**:

A mixture of equimolar amounts of the appropriate **7a**, **b**, **c**, **f** and the corresponding secondary amine (2 mmol) in dry acetonitrile (20 ml) containing potassium carbonate (4 mmol) was refluxed for 12h and the reaction mixture was filtered hot. The solid which separated upon storing the clear reaction mixture at room temperature overnight, was collected and crystallized from the suitable solvent.

*2-(4-(2-Chlorophenyl)piperazin-1-yl)-N-(4-oxo-2-(phenoxymethyl)quinazolin-3(4H)-yl)acetamide* (**8a**): White powders from ethanol; m.p. 206-207 ^o^C; yield 57%; IR ν/cm^-1^: 3200, 1680, 1590, 1550; ^1^H-NMR δ/ppm (CDCl_3_): 3.01-3.28 (m, 10H, piperazinyl and CH_2_), 5.03 (s, 2H, CH_2_O), 6.97-7.77 (m, 12H, arom-H), 8.10 (s, 1H, NH (D_2_O exchange)), 8.25 (d, *J*=7.65 Hz, 1H, arom-H_5_). Ms: m/z (%) 506 (M+2, 0.18), 504 (M, 0.46), 251 (C_15_H_11_N_2_O_2_, 4), 209 (C_11_H_14_ClN_2_, 100). Anal. for C_27_H_26_ClN_5_O_3_ (503.98): C, 64.35; H, 5.20; N, 13.90%. Found: C, 64.50; H, 5.60; N, 13.72%. 

*N-(2-((2-Chlorophenoxy)methyl)-4-oxo-quinazolin-3(4H)-yl)2-(4-(2-chlorophenyl) piperazin-1-yl)- acetamide* (**8b**): White crystals from ethanol; m.p. 159-160 ^o^C; yield 64%; IR ν/cm^-1^: 3177, 1679, 1616, 1585; ^1^H-NMR δ/ppm (CDCl_3_): 2.71-3.08 (m, 8H, piperazinyl H), 3.31 (s, 2H, CH_2_), 5.06 (d, *J*=12.2 Hz,1H, upfield proton of CH_2_O), 5.15 (d, *J*=12.6 Hz, 1H, downfield proton of CH_2_O), 6.91-7.80 (m, 12H, arom-H and NH), 8.25 (d, *J*=7.80 Hz, 1H, arom-H_5_); Anal. calcd. for C_27_H_25_Cl_2_N_5_O_3_ (538.42): C, 60.23; H, 4.68; N, 13.01%. Found: C, 59.90; H, 4.95; N, 12.80%. 

*N-(2-((2-Chlorophenoxy)methyl)-4-oxo-quinazolin-3(4H)-yl)-2-(4-methylpiperazin-1-yl)acetamide* (**8c**): White crystals from ethanol; m.p. 118-120 ^o^C; yield 52%; IR ν /cm^-1^: 3450, 1680, 1620, 1600; ^1^H-NMR δ/ppm (CDCl_3_): 2.26 (s, 3H, CH_3_), 2.48-2.80 (m, 8H, piperazinyl H), 3.19 (s, 2H, CH_2_), 5.04 (d, *J*=11.8 Hz ,1H, upfield proton of CH_2_O), 5.12 (d, *J*=11.2 Hz, 1H, downfield proton of CH_2_O), 6.95-7.80 (m, 8H, arom-H and NH), 8.27 (d, *J*=7.80 Hz, 1H, arom-H_5_) ; Anal. calcd. for C_22_H_24_ClN_5_O_3_ (441.91): C, 59.79; H, 5.47; N, 15.85%. Found: C, 59.64; H, 5.80; N, 15.73%. 

*2-(4-(2-Chlorophenyl)piperazin-1-yl)-N-(2-((2,4-dichlorophenoxy)methyl)-4-oxo-quinazolin-3(4H)-yl) acetamide* (**8d**): White crystals from ethanol; m.p. 188-189 ^o^C; yield 57%; IR ν/cm^-1^: 3171, 1674, 1609, 1585; ^1^H-NMR δ/ppm (CDCl_3_): 2.79-3.14 (m, 8H, piperazinyl H), 3.37 (s, 2H, CH_2_), 5.08 (d, *J*=12.2 Hz,1H, upfield proton of CH_2_O), 5.18 (d, *J*=12.0 Hz, 1H, downfield proton of CH_2_O), 6.98-7.88 (m, 11H, arom-H and NH), 8.27 (d, *J*=8.0 Hz,1H, arom-H_5_) ; Anal. calcd. for C_27_H_24_Cl_3_N_5_O_3_ (572.87): C, 56.61; H, 4.22; N, 12.23%. Found: C, 56.50; H, 4.11; N, 12.42%. 

*3-(4-(2-Chlorophenyl)piperazin-1-yl)-N-(2-((2,4-dichlorophenoxy)methyl)-4-oxo-quinazolin-3(4H)-yl) propamide* (**8e**): White crystals from ethanol; m.p. 205-206 ^o^C; yield 68%; IR ν/cm^-1^: 3390, 1702, 1611, 1585; ^1^H-NMR δ/ppm (CDCl_3_): 2.66 (t, *J*=5.2 Hz, 2H, CH_2_Cl), 2.81-3.10 (m, 10H, piperazinyl H and CH_2_CO), 4.97 (d, *J*=16 Hz,1H, upfield proton of CH_2_O), 5.23 (d, *J*=12.8 Hz, 1H, downfield proton of CH_2_O), 6.95-7.78 (m, 11H, arom-H and NH), 8.23 (d, *J*=8.2 Hz, 1H, arom-H_5_); Anal. calcd. for C_28_H_26_Cl_3_N_5_O_3_ (585.11): C, 57.30; H, 4.47; N, 11.95%. Found: C, 57.28; H, 4.46; N, 11.83%. 

### Synthesis of 3-((substituted methylamino)-2-(un/substituted phenoxymethyl) quinazolin- 4(3H)-one **9a,b**:

A mixture of formaldehyde (37-41%, 1 mL) and the appropriate amines (5 mmol) in dry DMF (5 mL) was added dropwise with stirring to a solution of **5** (5 mmol) in dry DMF (5 mL). The reaction mixture was filtered while hot and heated in a boiling water bath for 30 min. After cooling, it was poured onto crushed ice and the resulting solid was filtered, washed with water and crystallized from methanol.

*3-((4-(2-Chlorophenyl)piperazin-1-yl)methylamino)-2-(phenoxymethyl)quinazolin- 4(3H)-one* (**9a**): Off-white powder from methanol; m.p. 171-172 ^o^C; yield 74%; IR ν/cm^-1^: 3220, 1670, 1600, 1550; ^1^H-NMR δ/ppm (CDCl_3_): 2.76-3.435 (m, 8H, piperazinyl H), 3.94 (s, 2H, CH_2_), 5.13 (d, *J*=11.6 Hz,1H, upfield proton of CH_2_O), 5.18 (d, *J*=11.0 Hz,1H, downfield proton of CH_2_O,), 6.94-7.56 (m, 13H, arom-H and NH), 8.22 (d, *J*=7.6 Hz, 1H, arom-H_5_); Anal. calcd. for C_26_H_26_ClN_5_O_2_ (475.99): C, 65.61; H, 5.51; N, 14.71%. Found: C, 65.90; H, 5.10; N, 14.77%. 

*2-((2,4-Dichlorophenoxymethyl)-3-((4-methoxyphenylamino)methylamino)quinazolin-4(3H)-one* (**9b**): Off-white powder from methanol; m.p. 212-214 ^o^C; yield 68%; IR ν/cm^-1^: 3250, 1670, 1580, 1560; ^1^H-NMR δ/ppm (CDCl_3_): 3.78 (s, 3H, OCH_3_), 4.82 (s, 2H, CH_2_), 5.21 (d, *J*=12.2 Hz, 1H, upfield proton of CH_2_O), 5.32 (d, *J*=13.6 Hz, 1H, downfield proton of CH_2_O,), 6.83-7.81 (m, 12H, arom-H and 2NH), 8.30 (d, *J*=8.0 Hz,1H, arom-H_5_,); Anal. calcd. for C_23_H_20_Cl_2_N_4_O_3_ (471.33): C, 58.61; H, 4.28; N, 11.89%. Found: C, 58.13; H, 4.00; N, 11.79%. 

### Anticonvulsant activity

Albino mice (purchased from the National Research Centre Animal House) weighing 20-25 gm were kept under hygienic conditions and on standard laboratory diet (diet composition A. O. A. C.: vitamin mix. 1%, mineral mix. 4%, sucrose 20%, cellulose 0.2%, 5% pure casein 10.5%, starch 54.3%) and water was provided *ad libitum*. Mice were divided into groups of six animals each. The treated groups received the tested compounds intraperitoneally in a dose of 40 mg/kg body weight in DMSO while the control group received DMSO. The standard group received diazepam in a dose of 5 mg/kg. One hour after the injection, electroshock was applied via ear-lip electrodes and generated by a stimulator (Ugo Basile ECT Unit, Pulse generator 57800-001, delivering an alternating 50 Hz current), the stimulus duration was 0.2 second and the end point was tonic hind limb extension. The maximum electro-shock was determined. Then, % protection as well as % potency were calculated according to the following equations:

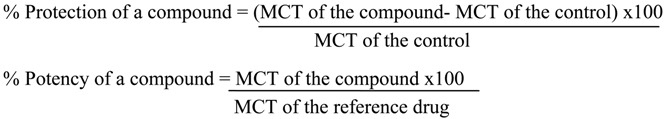

where MCT = Mean Convulsion Threshold
